# A multidimensional stability model for predicting shallow landslide size and shape across landscapes

**DOI:** 10.1002/2014JF003135

**Published:** 2014-11-26

**Authors:** David G Milledge, Dino Bellugi, Jim A McKean, Alexander L Densmore, William E Dietrich

**Affiliations:** 1 Department of Geography, Durham UniversityDurham, UK; 2 Department of Earth Atmospheric and Planetary Science, Massachusetts Institute of TechnologyCambridge, Massachusetts, USA; 3 U.S. Department of Agriculture, Forest Service, Rocky Mountain Research StationBoise, Idaho, USA; 4 Department of Earth and Planetary Science, University of CaliforniaBerkeley, California, USA

**Keywords:** shallow landslides, landslide size, depth-area scaling, slope stability model

## Abstract

The size of a shallow landslide is a fundamental control on both its hazard and geomorphic importance. Existing models are either unable to predict landslide size or are computationally intensive such that they cannot practically be applied across landscapes. We derive a model appropriate for natural slopes that is capable of predicting shallow landslide size but simple enough to be applied over entire watersheds. It accounts for lateral resistance by representing the forces acting on each margin of potential landslides using earth pressure theory and by representing root reinforcement as an exponential function of soil depth. We test our model's ability to predict failure of an observed landslide where the relevant parameters are well constrained by field data. The model predicts failure for the observed scar geometry and finds that larger or smaller conformal shapes are more stable. Numerical experiments demonstrate that friction on the boundaries of a potential landslide increases considerably the magnitude of lateral reinforcement, relative to that due to root cohesion alone. We find that there is a critical depth in both cohesive and cohesionless soils, resulting in a minimum size for failure, which is consistent with observed size-frequency distributions. Furthermore, the differential resistance on the boundaries of a potential landslide is responsible for a critical landslide shape which is longer than it is wide, consistent with observed aspect ratios. Finally, our results show that minimum size increases as approximately the square of failure surface depth, consistent with observed landslide depth-area data.

## 1. Introduction

Shallow landslides usually involve only the colluvial soil mantle and are generally translational, failing along a quasi-planar surface. They are important as agents of landscape-scale sediment transfer and erosion as well as potential hazards to life and infrastructure [*Spiker and Gori*, 2003]. The importance of each landslide is defined by its location and size.

**Figure 1:**
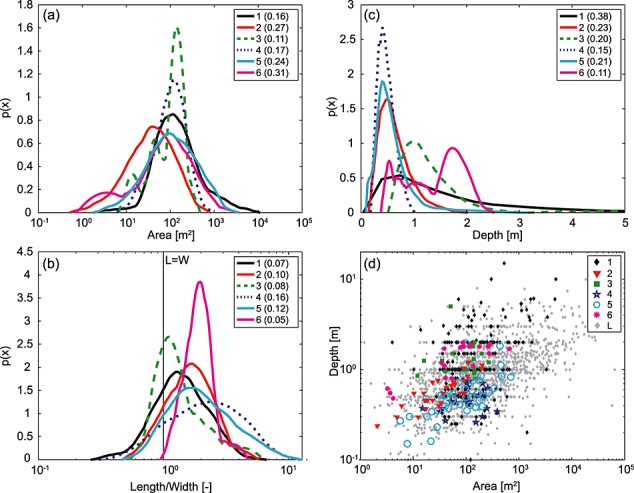
Observed landslide properties from six published inventories showing empirical PDFs of (a) landslide scar area, (b) scar length (*L*) to width (*W*) ratio, (c) scar depth, and (d) a scatter plot showing the power relationship between scar depth and area. The inventories are from (1) the Appalachian mountains [*Morgan et al*., 1997]; (2) Hakoishi, Japan [*Paudel et al*., 2003]; (3) San Gabriel Mountains, California [*Rice et al*., 1969]; (4) Santa Barbara County, California [*Gabet and Dunne*, 2002]; (5) Cumbria, England [*Warburton et al*., 2008]; and (6) Oregon Coast Range [*Montgomery*, 1991; *Larsen et al*., 2010]. Grey diamonds in Figure 1d are the scar dimensions for soil landslides from a global compilation by *Larsen et al*. [2010]. PDFs are generated using kernel density functions after *Epanechnikov* [1969], with optimized half widths given in brackets in each legend.

While much progress has been made in mechanistic prediction of landslide location [e.g., *Montgomery and Dietrich*, 1994; *Casadei et al*., 2003a; *Tarolli and Tarboton*, 2006; *Baum et al*., 2010; *Lanni et al*., 2012] we remain limited in our understanding of what controls landslide size (area and depth), which is fundamental to both hazard [*Hungr et al*., 2008] and geomorphic change [*Dietrich et al*., 2008]. Field mapped inventories of shallow landslides (Figure 1) [*Rice et al*., 1969; *Montgomery*, 1991; *Morgan et al*., 1997; *Gabet and Dunne*, 2002; *Paudel et al*., 2003; *Warburton et al*., 2008; *Larsen et al*., 2010] show that their scar size varies across several orders of magnitude in volume (10^0^–10^5^ m^3^) and area (10^1^–10^4^ m^2^). All six inventories have clear modes (Figure 1a) and 70% of the scar areas are between 30 and 300 m^2^. The landslides are generally longer than they are wide (Figure 1b; *L* > *W* for 70–100% of landslides), and wider than they are deep (*W* > *D* for 99% of landslides). Since the landslides are generally restricted to the soil mantle they rarely extend beyond a few meters deep, and the majority are between 0.1 and 1 m deep (Figure 1c). Landslide depth appears to scale as a power function of surface area both for some individual inventories (Figure 1d) and for global compilations of soil and bedrock landslides, albeit with almost 2 orders of magnitude of scatter in the global compilation [*Guzzetti et al*., 2009; *Larsen et al*., 2010].

Why do shallow landslide depth and area distributions have these characteristics, and why does landslide depth scale with area? Why are landslides longer than they are wide and wider than they are deep?

An absolute upper limit to size is defined by hillslope length and width, which limit the area of the soil mantle that can fail as a single body [*Frattini and Crosta*, 2013]. In practice the upper limit is considerably smaller and is likely to relate to the spatial extent of low-strength areas [*Pelletier et al*., 1997; *Frattini and Crosta*, 2013; *Alvioli et al*., 2014]. Soil thickness sets an upper limit on shallow landslide depth, and most shallow landslides fail at the base of the colluvial soil where typically the permeability decreases and strength increases [*Larsen et al*., 2010]. There is a theoretical lower limit to both landslide depth and area in cohesive material because a landslide must be large enough for its driving force to overcome the constant stress-independent cohesion on its failure surface. This has been demonstrated for a range of depth-varying cohesion fields representative of soil and rock [*Frattini and Crosta*, 2013]; as well as root cohesion, which dominates in many colluvial soils [*Reneau and Dietrich*, 1987; *Casadei et al*., 2003b; *Gabet and Dunne*, 2002; *Dietrich et al*., 2008].

Between these limits to landslide depth, *Dietrich et al*. [2008] and *Frattini and Crosta* [2013] have shown that frictional resistance and cohesion on the margins of a landslide interact to create a least stable depth that can be within rather than at the base of the soil profile. Distributions of scar area have been explained by: the dynamics of rupture propagation [*Piegari et al*., 2006; *Stark and Guzzetti*, 2009; *Lehmann and Or*, 2012] or the distribution of low-strength patches [*Pelletier et al*., 1997; *Katz and Aharonov*, 2006; *Frattini and Crosta*, 2013; *Alvioli et al*., 2014]. The presence of cohesion is essential to almost all these explanations; in its absence, the controls on the lower limit to landslide depth and area have not been identified.

*Klar et al*. [2011] used a two-dimensional analytical model to give the first mechanistic explanation for the observed scaling relationship between landslide depth and area (Figure 1d). Applying the model to slopes of varying length, they found that depth scaled as approximately the square root of landslide length, where landslide length is defined by slope length and depth is the free parameter. This can be used to reproduce the square root dependence of landslide depth on area under the following assumptions: (1) that landslide width is a linear function of length, and (2) that either landslide length is always constrained and depth the free parameter or the modeled length-depth relationship can be inverted to predict length when depth is constrained and length the free parameter [*Klar et al*., 2011].

Observed landslide length and width almost always exceed depth, with depths generally less than 2 m and areas greater than 4 m^2^ [*Larsen et al*., 2010]. It is commonly acknowledged that length exceeds width [e.g., *Gabet and Dunne*, 2002; *Rickli et al*., 2008; *Marchesini et al*., 2009]. However, very few studies have attempted to explain this behavior. *Lehmann and Or* [2012] were able to reproduce, but not explain, the general behavior of length and width using a fiber bundle model to represent progressive failure but suggested that their results were strongly dependent on model choices as well as local heterogeneities.

We aim (1) to examine whether resistances on the margins of a landslide influence its length and width, (2) to extend the existing theory on lower limits to landslide depth and area from cohesive soils into cohesionless soils, and (3) to develop an alternative physically based explanation for the observed landslide depth-area scaling. To do this, we need a slope stability model that can test the stability of potential landslides of varying three-dimensional geometries with different material properties and that is suitable for application to natural slopes. Since none of the currently available stability models fully satisfy these requirements (see review in section 2), we derive a new model that retains the low data requirements of existing models but is more faithful to the key processes that control the stability of natural slopes (section 3). We demonstrate the implications of the new analysis in section 4 then test it for an observed landslide where the parameters are well constrained by field measurement (section 5). Finally, we apply the model to identify the physical mechanisms that explain the observations above.

## 2. Existing Slope Stability Models

Most slope stability models perform a limit equilibrium analysis for a defined failure surface, assuming that stresses are uniformly mobilized over the whole failure surface and that the soil mass behaves as one or more rigid blocks. Shallow landslide models almost exclusively use the simplest form of this analysis: the one-dimensional infinite slope equation [*Haefeli*, 1948; *Taylor*, 1948; *Skempton and DeLory*, 1957] coupled with a hydrological model to estimate the local pore pressure field [e.g., *Montgomery and Dietrich*, 1994; *Iverson*, 2000; *Casadei et al*., 2003a; *Tarolli and Tarboton*, 2006; *Baum et al*., 2008; *Lanni et al*., 2012]. However, understanding landslide size and shape requires a three-dimensional model where the dimensions of the landslide can be examined explicitly and where the resistance on the margins of a potential landslide can be represented.

The simplest three-dimensional approaches consider the forces acting on a single block in limiting equilibrium and treat either lateral root reinforcement [*Burroughs*, 1985; *Reneau and Dietrich*, 1987; *Montgomery et al*., 2000; *Gabet and Dunne*, 2002; *Casadei et al*., 2003b] and/or boundary pressure on the margins of the block [*Chen*, 1981; *Burroughs*, 1985]. When included, root reinforcement is generally treated as an effective cohesion. Boundary pressures are modeled using earth pressure theory and assuming an active wedge upslope of the block (driving failure), a passive wedge downslope (resisting failure), and pressure on the cross-slope sides generating shear resistance due to friction. With the exception of *Burroughs* [1985], the upslope and downslope wedges were assumed to be horizontal (i.e., earth pressure coefficients depended only on soil friction angle). Furthermore, cohesion is either ignored or represented as an additive term on the upslope and downslope boundaries, rather than acting on the wedges themselves. This is particularly problematic in the downslope case where the soil is failing under compression.

An alternative approach is to extend the two-dimensional method of slices [e.g., *Morgenstern and Price*, 1965; *Spencer*, 1967] into the third dimension, discretizing the landscape into columns [e.g., *Hovland*, 1977; *Lam and Fredlund*, 1993]. However, these methods do not consider shear resistance (due to friction or cohesion) on the cross-slope boundary between stable and unstable columns and so underestimate shear resistance on that boundary [*Stark and Eid*, 1998; *Chugh*, 2003].

*Dietrich et al*. [2008] applied a framework similar to *Hovland*'s [1977] method, but parameterized the forces on the margins of the landslide using methods similar to *Burroughs* [1985]. *Dietrich et al*. [2008] assumed horizontal upslope and downslope wedges to enable an analytical solution but inclined the resultant forces by the soil friction angle to represent friction on the margin between the blocks. As in *Burroughs* [1985] they assumed that the upslope and downslope wedges are cohesionless, and then added cohesion to each of the block's vertical boundaries.

In a limit equilibrium analysis, all forces are assumed to occur at the same instant. However, some slides may develop incrementally with a small area failing first and its load then being transferred to neighboring areas, causing them to fail. This style of progressive failure is normally modeled using a Finite Element Model [*Duncan*, 1996; *Griffiths and Marquez*, 2007], but *Lehmann and Or* [2012] attempted to approximate progressive failure in a limit equilibrium framework by treating deformation implicitly using rigid columns, but removing and re-distributing the load of each column once it had failed by basal shear. They represented the driving and resisting forces acting on the basal, upslope, downslope, and cross-slope margins of each column, focusing on cohesive effects. They did not represent friction on the cross-slope margin and an upslope cell only exerts a driving force on its downslope neighbors once it has failed at its base. They represented the critical downslope stress required to cause failure using a water-dependent compression strength threshold [*Mullins and Panayiotopoulos*, 1984] that does not account for the self-weight of the soil, which is appropriate for unconfined samples but not for natural slopes.

These models have enabled analysis of discrete landslides within a limit equilibrium framework and are capable of representing the lateral forces acting on a potential landslide, which is essential for a three-dimensional treatment. However, they are generally limited by their treatment of upslope and downslope margins, either assuming that the ground surface is horizontal above and below the landslide, incorrectly accounting for cohesion on these margins or neglecting the self-weight of the soil. This is a problem because the forces acting on these margins can strongly affect both the stability of the slope and the geometry of the landslide. To address this problem, we extend the method presented by *Dietrich et al*. [2008], relaxing the assumption that the upslope and downslope wedges have a horizontal surface, and include the effect of cohesion on their failure surface (i.e., earth pressure coefficients depend on friction angle, slope, and cohesion). This approach retains the simplicity and analytical tractability of standard limit equilibrium approaches but is a more faithful representation of natural slope conditions.

## 3. The Multidimensional Shallow Landslide Model

**Figure 2:**
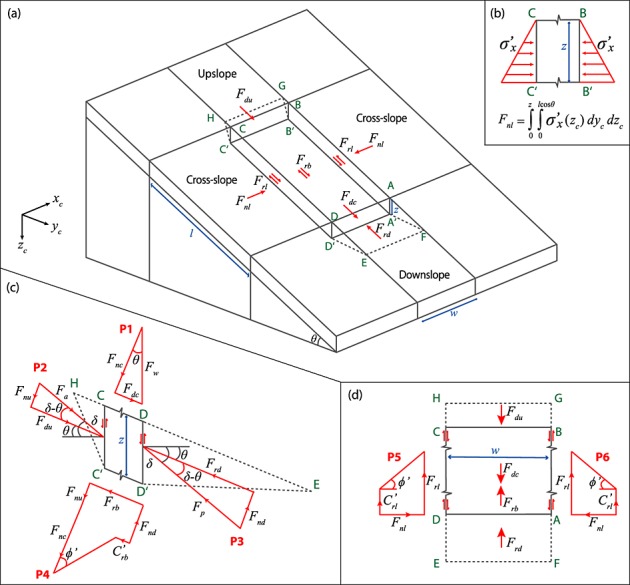
Schematic showing forces and lengths for the three-dimensional slope stability problem in (a) 3-D, (b) cross section, (c) profile, and (d) plan. MD-STAB computes the stability of a potential landslide by calculating the forces on each of the planes shown here. The red arrows in Figures 2^2^a–2^2^d show the forces acting on each margin of the block. The red arrows in Figure 2b show the stress distribution on the cross-slope sides of the block. Red force polygons P1–P6 in Figures 2c and 2d illustrate the magnitude and orientation of forces acting on the block and their combination (i.e., vector sum) to give resultant forces: (P1) normal and driving forces on the central block; (P2) active force on the upslope margin; (P3) passive force on the downslope margin; (P4) normal and resisting forces on the base central block; and (P5) and (P6) normal and resisting forces on the cross-slope sides.

The MD-STAB model satisfies horizontal and vertical force equilibrium while ignoring moment equilibrium. A shallow landslide is represented by three connected three-dimensional hillslope segments: an active (upslope) wedge, a central block and a passive (downslope) wedge. A force balance is calculated on the central block. Figure 2 shows the geometry of the three segments and force polygons which illustrate the magnitude and orientation of the forces acting on the central block. The central block is assumed to be rigid and to fail by shear on a plane parallel to the ground surface at a prescribed depth. Typically, this plane is the soil-bedrock interface, which is often the location of the largest contrast in material strength in hillslope soils. We also explore the influence of failure plane depth on the stability and size of a potential landslide. We assume that failure occurs in drained conditions and that groundwater flow is steady and parallel to the slope surface, although other groundwater assumptions could also be used to predict a pore water pressure field. We also ignore any infiltration, suction, or capillary rise effects in an unsaturated zone and simply partition the landslide block into saturated and unsaturated zones. This allows definition of a saturation ratio (*m* = *h*/*z*) where *h* is the height of the water table and *z* is the depth to the failure surface. Driving forces include the downslope component of the central block mass plus the force on the central block from the upslope wedge where active earth pressure conditions are assumed. Resisting forces are considered on all boundaries of the central block, and include the passive earth pressure from the downslope wedge and soil friction and root cohesion on the base, cross-slope, upslope, and downslope sides. Cohesion is not directly added at the upslope and downslope vertical boundaries of the central block. Instead, resistance due to cohesion is incorporated in the passive and active wedges themselves and affects the corresponding earth pressures that those wedges impose on their boundaries with the central slide block. The following sections describe in detail the conceptualization of the driving and resisting forces internal and external to the central slide block, and how the forces are combined to evaluate the factor of safety of a potential landslide.

### 3.1. Central Block Driving Force (*F*_dc_)

This model component represents the driving force caused by the mass of the slide block itself. We follow closely the standard formulation used in other plane strain landslide analyses, such as a method of slices or the infinite slope method, but eventually calculate a driving force, rather than stress, for a finite three-dimensional slide (Figure 2a). The total vertical geostatic stress *σ_z_* at depth *z* caused by the soil above it is


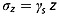
(1)

where *γ_s_* is the unit weight of the soil (*γ_s_* = *g ρ_s_*), *ρ_s_* is the constant bulk density of soil and *g* is gravitational acceleration. Note that in common with many other studies [e.g., *Montgomery and Dietrich*, 1994; *Iverson*, 2000; *Gabet and Dunne*, 2002; *Baum et al*., 2008; *Lanni et al*., 2012], we assume a single soil density independent of soil moisture content since it ultimately has very little impact on the computed factor of safety. The driving component of this stress *τ* acts downslope along the failure surface and is


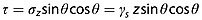
(2)

where *θ* is the slope inclination (Figure 2). The corresponding driving force *F*_dc_ acting downslope along the failure surface is the driving stress integrated over the planimetric length and width of the slide (polygon P1 in Figure 2c):


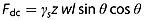
(3)

where *l* is the slide length and *w* is its width (Figure 2).

### 3.2. Block Cross-Slope Boundaries (*F*_rl_)

Shear resistance on the two parallel and vertical cross-slope sides of the slide block *F*_rl_ results from friction and cohesion. These sides are the surfaces ABB′A′ and DCC′D′ in Figure 2a, and the forces acting on them are shown by polygons P5 and P6 in Figure 2d. Following *Stark and Eid* [1998], we represent friction by assuming that external horizontal and vertical forces act at the centers of the two sides and that these forces can be predicted from standard earth pressure theory. For a homogeneous soil with isotropic frictional properties, the shear resistance due to lateral earth pressure on the cross-slope sides is the product of the horizontal stress at a point and the soil friction angle (polygons P5 and P6 in Figure 2d). We assume that in the cross-slope direction, earth pressure in the soil layer is in an intermediate or “at-rest” condition (i.e., it does not experience active or passive yield during failure). The at-rest lateral earth pressure *σ*′*_x_* at any point is conventionally calculated from the vertical effective pressure *σ*′*_z_* as


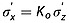
(4)

where *K*_0_ is the coefficient of at-rest earth pressure. Since *σ*′*_z_* increases linearly with depth, *σ*′*_x_* has the triangular stress distribution shown in Figure 2b. The at-rest earth pressure coefficient is poorly constrained for soils with cohesion. In particular, when roots contribute to this cohesion they may support some of the vertical geostatic stress reducing the value of *K*_0_, but this effect is difficult to quantify. As a result, most applications use *Jaky*'s [1944] empirical formula for cohesionless, normally consolidated soils, commonly found on natural slopes [*Das*, 2009]. Under these assumptions the at-rest earth pressure coefficient is


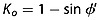
(5)

where *φ*′ is the effective friction angle of the soil. The cross-slope resisting stress *s_c_* on the vertical sides of the slide block is


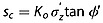
(6)

To calculate the resisting force *F*_rc_ on a cross-slope boundary, we integrate equation (6) over the cross-slope area of the slide block, (*l z cos θ*) and add the depth averaged cohesion *C*'_rl_ acting over the same area (polygons P5 and P6 in Figure 2d):


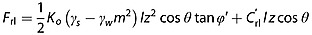
(7)

where *γ_w_* is the unit weight of water, *m* is the saturation ratio (*m* = *h*/*z*), and *h* is the height of the water table above the failure surface.

### 3.3. Block Upslope (*F*_du_) and Downslope (*F*_rd_) Boundaries

For a landslide to occur, i.e., for shear to develop on the base of the central block, the downslope wedge must fail and mobilize under passive or compressive earth pressure conditions. At the same time, the failing central block will move away from the soil upslope of it, creating active or tensile conditions in the upslope wedge. We model the interfaces between these wedges and the central block as vertical boundaries (see the surfaces BCC′B′ and ADD′A′ in Figure 2a). The effects of these two soil wedges on the central block are calculated from the active *F_a_* and passive *F_p_* forces that they impose on these upslope and downslope vertical boundaries (polygons P2 and P3, in Figure 2c). The active and passive forces are defined using standard earth pressure theory (e.g., used to analyze retaining wall stability), but including cohesion in the upslope/downslope wedges and an inclined soil layer appropriate for natural slopes.

Classical soil mechanics theory includes three primary methods of active and passive earth pressure prediction, the Rankine, Coulomb, and log-spiral methods, which are described in standard soil mechanics textbooks [e.g., *Das*, 2009]. All three methods assume a homogeneous and isotropic soil. The *Rankine* [1857] method is a lower bound plasticity solution based on statically admissible stress fields, while the *Coulomb* [1776] and log-spiral methods [*Caquot and Kerisel*, 1948; *Chen*, 1975] are upper bound solutions based on kinematically admissible velocity fields [*Das*, 2009]. The three methods have also been modified to allow for a sloping soil layer and cohesive soil [*Chugh and Smart*, 1987; *Mazindrani and Ganjali*, 1997; *Gnanapragasam*, 2000; *Soubra and Macuh*, 2002].

These earth pressure theories primarily differ in how they treat stress conditions on their boundaries with the central block and how they model the failure surface beneath the wedges. During failure the upslope block will tend to move vertically downward along the interface with the central block as they both translate downslope. This introduces a downward shear along the upslope boundary of the central block that reorients the resultant active force by some angle *δ* from horizontal (polygon P2 in Figure 2c). On the downslope passive interface, shear develops in the opposite sense and again the passive force is reoriented from horizontal (polygon P3 in Figure 2c). The Rankine method assumes that the force reorientations are equal to the slope angle (i.e., *δ* = *θ*), while *δ* can take any value from 0 to φ′ in the Coulomb and log-spiral methods [*Duncan and Mokwa*, 2001]. The Rankine and Coulomb methods assume that the failure surfaces beneath the active and passive wedges are planar, but theory and observation demonstrate that they are curved [*Terzaghi*, 1967]. In the active case the curvature is small and a planar assumption causes little error [*Craig*, 2004]. But in the passive case, a planar failure surface results in passive pressure predictions that are much too large, particularly if *δ* > 0.4 *ϕ*′ [*Duncan and Mokwa*, 2001]. *Terzaghi* [1967] described a failure surface that took the form of the arc of a logarithmic spiral and passive earth pressure predictions using this wedge geometry were found to be more accurate over any value of *δ* [*Soubra*, 2000; *Zhu and Qian*, 2000]. However, this requires optimizing the two parameters that describe the failure surface for each combination of slope, friction angle, cohesion, and soil thickness.

Because of the uncertainty in analytical predictions of the active and passive forces on the central block, we calculate lower and upper bounds. To obtain a lower bound estimate, we use the Rankine method at both margins. To obtain an upper bound estimate, we use the log-spiral method, which can allow for curvature on the failure surface, at the downslope margin and the simpler Coulomb method at the upslope margin, which is typically planar. Following *Mazindrani and Ganjali* [1997], the Rankine solution for cohesive soils on a hillslope gives the lower bound active, *K_a_* and passive, *K_p_* earth pressure coefficients:


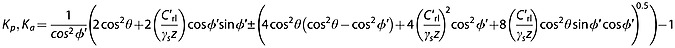
(8)

where *φ*′ is the soil friction angle, *θ* is the slope angle, *C*′_rl_ is the depth averaged cohesion, *γ_s_* is the soil unit weight, *z* is the depth of the failure plane of the central block, and the negative and positive signs are for the active and passive cases, respectively. Following *Chugh and Smart* [1987], the Coulomb active earth pressure coefficient *K_a_* for sloping cohesive soils is defined as


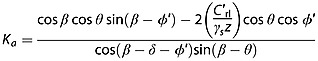
(9)

where *β* is the inclination from horizontal of a planar failure surface from the base of the central block to the ground surface upslope. We solve equation (9) numerically to find the most critical failure plane (for *θ* − 1° < *β* < 89°) which maximizes the active earth pressure coefficient [*Chugh and Smart*, 1987].

We use the version of the log-spiral method derived by *Soubra and Macuh* [2002] to provide an upper bound solution for the passive resistance of sloping cohesive soils downslope of a potential slide mass. *Soubra and Macuh* [2002] employed a rotational logarithmic spiral failure surface on the basis that under these conditions an energy balance is equivalent to moment equilibrium about the center of the logarithmic spiral. The solution requires identification of the most critical log-spiral failure plane (i.e., minimizing passive pressure), and yields


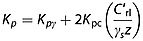
(10)

where *K_pγ_* and *K_pc_* are the friction and cohesion components of the passive earth pressure coefficient, respectively. The passive earth pressure coefficient is thus a function of slope, friction angle, cohesion, soil unit weight, soil depth, and two geometry parameters *α*_0_ and *α*_1_, which define the geometry of the logarithmic-spiral failure surface (full equations provided in Appendix A). Following *Soubra and Macuh* [2002], we solve equation (10) numerically using a generalized reduced gradient algorithm [*Lasdon et al*., 1978] to find the log-spiral failure surface that minimizes the passive earth pressure coefficient.

By treating the upslope and downslope margins as analogous to the wall in an earth pressure retaining wall problem the active *σ_a_* and passive *σ_p_* stresses on the upslope or downslope margin of the central block can be calculated as the product of the vertical effective pressure (*σ*′*_z_*) and the active or passive earth pressure coefficients from equations (8), or (10). For the passive downslope margin


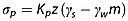
(11)

To calculate the total passive force on the downslope margin (ADD′A′ in Figure 2a) we integrate equation (11) over the downslope boundary of the block (*wz*) perpendicular to the direction of sliding. This passive force *F*_*p*,_ is the resultant of both the normal and shear forces (due to friction) on the boundary between the central block and the wedge and is inclined at the boundary friction angle *δ*. We assume that *δ* = *θ*, in the lower bound case and *δ* = *ϕ*′ in the upper bound case. As a result, the passive force needs to be decomposed into its slope-parallel component, which acts as a resisting force *F*_rd_:


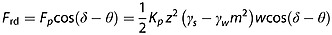
(12)and a slope normal component *F*_nd_, which modifies the normal force on the base of the central block (polygon P3 in Figure 2c):


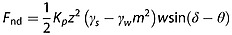
(13)

The active stress *σ_a_* on the upslope margin follows the same form as the passive stress and can be calculated from equation (11) by replacing the passive coefficient with an active earth pressure coefficient *K_a_* for sloping soils. The net driving force on the upslope margin *F*_du_ can then be calculated from equation (12) making the same substitution (Figure 2c). For soils with a strong cohesive component the active earth pressure coefficient, and therefore, the net driving force on the upslope margin, is negative since the resisting forces due to cohesion exceed the driving force of the upslope wedge. In this case the negative *F*_du_ represents a net resisting force on the upslope margin of the central block. Note that cohesion on the wedge failure surface is included within the active and passive earth pressure coefficients and does not need to be applied to the vertical upslope or downslope boundaries (equations (12) and (13)). The slope normal component of the active force *F*_nu_, which modifies the normal force on the base of the central block, can be calculated from equation (13) by replacing the passive with the active earth pressure coefficient (P2 in Figure 2c).

Standard earth pressure methods use a hydrostatic analysis to calculate earth pressure on the upslope and downslope boundaries of the unstable block [*Das*, 2009]. In reality, slope-parallel seepage will exert a force on these boundaries increasing the driving force on the upslope boundary and reducing passive resistance on the downslope boundary. However, to our knowledge, there is currently no suitable earth pressure method that can account for seepage forces in the upslope active wedge and downslope passive wedge. We discuss the impact of this simplification on our findings in section 6.4.

### 3.4. Basal Resistance Force (*F*_rb_)

Resistance along the base of the slide block *F*_rb_ develops by a combination of cohesion *C*′_rb_ and friction, the product of normal force on the failure surface and the tangent of the friction angle. The normal force *F*_nt_ is the effective normal stress on the failure surface integrated over its area (thus accounting for pore pressure). It includes the normal force due to the self-weight of the central block (*F*_nc_, polygon P1 in Figure 2c), and the components of the upslope *F*_nu_ and downslope *F*_nd_ forces that act normal to the failure surface (polygon P4 in Figure 2c):


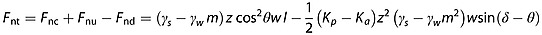
(14)

*F*_nd_ acts to decrease the normal force on the base of the central block when *δ* > *θ* and to increase it when *δ* < *θ*. The opposite is true of *F*_nu_; however, *F*_nu_ can also change sign in response to a negative active force at the upslope margin. Given this definition of the normal force on its base, the basal resistance force on the central block is then


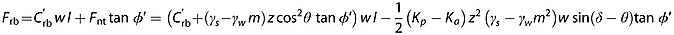
(15)

### 3.5. Complete Formulation

The Factor of Safety *FS* for the block can then be calculated as the ratio of driving to resisting forces by combining each of these components from equations (3), (7), (13), and (15):


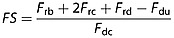
(16)

Substituting equations (3), (7), (13), and (15) into equation (16) and rearranging, the general form of the equation is


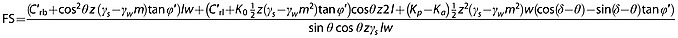
(17)

In the upper bound case we assume that *δ* = *ϕ*′ and equation (17) becomes


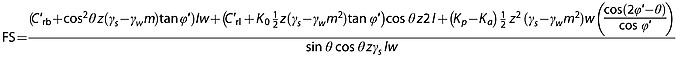
(18)

In the lower bound case we assume that *δ* = *θ* and equation (17) becomes


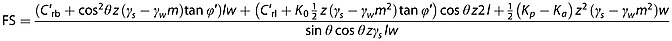
(19)

While these equations allow us to calculate the stability of a soil block, they do not include the variability in soil properties, slope geometry, and pore water pressure that occurs within an unstable hillslope, which is an important control on slope stability in natural landscapes. In the following section, we apply the same equations within a grid-based framework, which allows us to represent spatial variability in the model parameters.

### 3.6. Grid-Based Application

Following *Hovland* [1977], the normal and shear forces acting on the base of each column are derived as components of their weight and FS is calculated from the ratio of total available resistance to the total mobilized stress along the failure surface. As in *Hovland* [1977], we assume that there are no intercolumn shear forces within the group of columns that make up an unstable block. No progressive failure with strain softening, pore water pressure dynamics, or other unequal stress-strain behavior is considered. The resistive forces are applied to the outer boundary of the group of columns (i.e., the base and sides). Total resistance is the sum of these basal and lateral components (equations (7), (13) and (15)). The total driving force is the vector sum of the driving force vectors of each column within the potential landslide (equation (3)) and Figure 2). Since the grid is not oriented, slope-parallel most columns will have more than one force component (upslope, downslope, and cross-slope) acting on them. We decompose the lateral resistance on each column margin into its components by assigning a fraction of the edge length to each resistance component. For example, the upslope boundary of a grid cell that is oriented 30° from slope parallel will be assigned 63% upslope and 37% cross-slope resistance.

### 3.7. Parameterization of Cohesion

Cohesion acts on the base and lateral sides of a potential landslide and our model requires an assumption about the form of its variation with soil depth. Here we focus on colluvial slopes where the net soil cohesion is dominated by root strength [*Schroeder and Alto*, 1983; *Schmidt*, 1999]. Other forms of cohesion (e.g., due to cementation or suction) could easily be added given an expression for their variation with depth. Generally, root cohesion is not uniform with depth, as it is a function of root density, which typically declines exponentially with depth [e.g., *Roering*, 2008]. Following *Dunne* [1991] and *Benda and Dunne* [1997], we represent root cohesion as an exponential function of depth so that root cohesion on the basal failure plane *C*′_rb_ is defined as


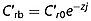
(20)

where *z* is failure plane depth, *C*′_*r*0_ is a coefficient representing the maximum root cohesion value at the surface, and *j* is an *e*-folding length scale. Root cohesion can be integrated over the block depth *z* (in the vertical coordinate *z_c_*) to obtain the average lateral root cohesion *C*′*_rl_* per unit perimeter area:


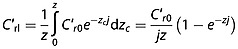
(21)

Equation (21) is applied to the cross-slope vertical boundaries and to the failure surfaces of the upslope and downslope wedges. When the downslope wedge failure surface is very curved this may result in a slight underestimation of cohesion on this boundary. This is a necessary approximation because the iterative method developed by *Soubra and Macuh* [2002] requires profile-averaged cohesion.

## 4. Significance of Model Assumptions

Estimated earth pressure coefficients can vary widely depending on which formulation is used to calculate them. In section 4.1 we compare our earth pressure coefficients with those that have previously been used in other stability models discussed in section 2. In section 4.2 we assess the relative contribution of friction and cohesion to lateral resistance on an example slope and examine the sensitivity of the resistive terms to slope geometry and material properties.

### 4.1. Effect of Different Earth Pressure Coefficients

**Figure 3:**
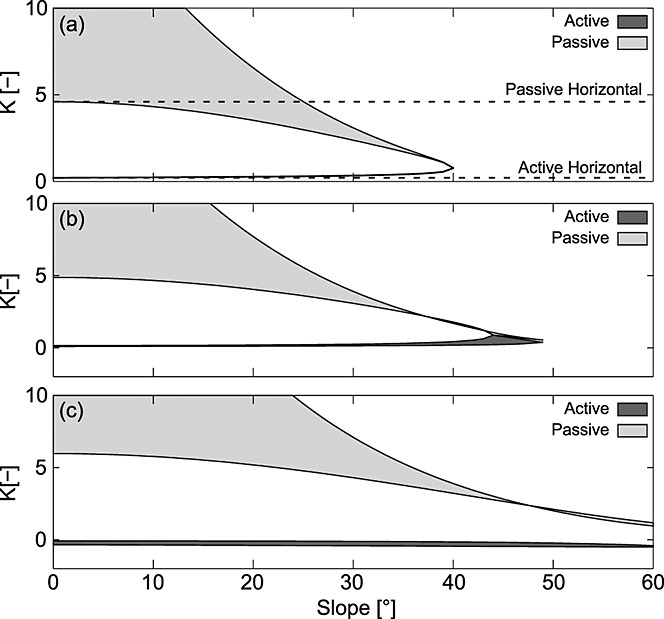
Earth pressure coefficients calculated using different methods. Parameter values used are z = 1; *θ* = 0–60°; *m* = 1; *φ*′ = 40°; *γ_s_* = 15.7 kN m^−3^; and (a) *C*′_rl_ = 0 kPa, (b) 1 kPa, and (c) 5 kPa. Shaded areas are defined by the upper and lower bound solutions. All lower bound solutions are derived using the Rankine method. Upper bound solutions for the active case at the head of a landslide are by the Coulomb method and for the passive case at the slide toe the solutions are by the log-spiral method. The horizontal coefficient results in an overestimate of passive resistance on steep slopes. The coefficients that account for sloping soils become indeterminate on cohesionless slopes greater than the friction angle. This problem is reduced by representing cohesion in the earth pressure coefficient (Figures 3b and 3c). Note that in this case the upper bound coefficient can fall below the lower bound coefficient at very high slopes suggesting that the treatment of earth pressure is approximate for slopes steeper than the friction angle.

Figure 3a shows the earth pressure coefficients in a cohesionless soil as a function of slope angle using different methods of prediction. The simplest formulation estimates earth pressure by assuming that the ground surface is horizontal in the upslope (active) and downslope (passive) wedges and that there is no friction on the boundaries between the wedges and the central block. In this classic soil mechanics approach [*Chen*, 1981; *Dietrich et al*., 2008], earth pressure is only dependent on the friction angle. Using both upper and lower bound methods, the active *K_ah_* and passive *K_ph_* coefficients of earth pressure are the familiar


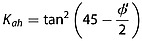
(22)


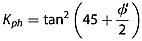
(23)and these pressures act perpendicular to the respective boundaries [*Das*, 2009].

Earth pressures predicted by the lower bound (Rankine) and upper bound (log-spiral and Coulomb) methods modified for sloping cohesionless soils illustrate the effects of slope angle (Figure 3a). The active pressure increases slightly at slopes between 38° and 40°, while the passive pressure declines sharply at steeper slopes until it equals the active pressure when the slope reaches the friction angle (here assumed to be 40°). The horizontal active earth pressure coefficient (*K_ah_*) agrees well with the modified upper and lower bound coefficients, although it results in a slight underestimation of the active earth pressure when the slope is >38°. The passive coefficient assuming a horizontal ground surface (*K*_ph_) falls between the upper and lower bound solutions for slopes gentler than 25° but results in a considerable overestimation of the passive earth pressure for slopes steeper than 25°, on which landslides are most likely.

On cohesionless slopes greater than the friction angle, earth pressure predictions become indeterminate for all the methods that account for sloping ground: the Rankine coefficients become complex because the square root term in equation (8) becomes negative; the Coulomb active coefficient goes to infinity because the failure surface that maximizes equation (9) becomes parallel with the slope and the active wedge becomes infinitely long; and the log-spiral slip surface degenerates to a planar surface with radii approaching infinity, violating the optimization constraints [*Soubra and Macuh*, 2002].

In practice, cohesionless soil is rarely found on slopes steeper than the friction angle, as some cohesion (provided by clay minerals, cementing agents, or more commonly vegetation roots) is usually necessary to maintain soil mass stability on steep slopes [*Das*, 2009]. Figures 3b and 3c show the earth pressure coefficients accounting for cohesion for two different scenarios: one where the cohesion is relatively low, representing weak roots (Figure 3b) such as have been measured in grasslands [*Buchanan et al*., 1990] and another where the cohesion is larger, but still modest, representing a more dense root network or stronger roots (Figure 3c) such as might be found in a forest [*Schmidt et al*., 2001]. Figure 3 shows that even a modest amount of additional cohesion considerably extends the range of slopes over which the earth pressure coefficients can be predicted. Figure 3 also shows that when cohesion is included in the earth pressure coefficient, the upper and lower bounds can cross at very high slopes suggesting that the treatment of earth pressure is approximate for slopes steeper than the friction angle. However, the bounds do not significantly diverge on high slopes, suggesting that the approximation is reasonable. In practice, shallow landslides are not common on these extreme slopes where a soil mantle is unlikely to persist in the absence of high cohesion.

### 4.2. Lateral Strength Contribution of Friction

**Figure 4:**
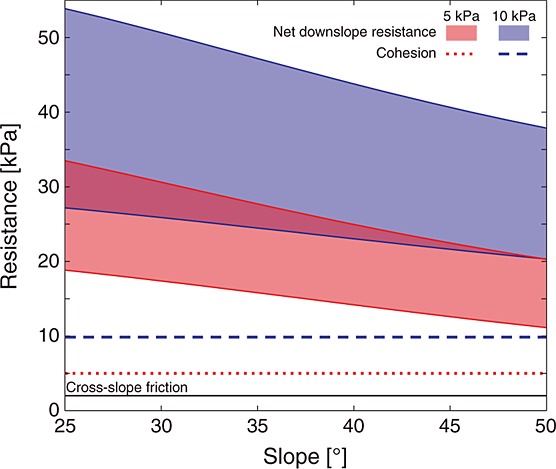
Lateral and net downslope resistances from different strength components at different slope angles for a block of soil with *γ_s_* = 15.7 kN m^−3^, *φ*′ = 40°, *z* = 1 m, and *m* = 0.5, for a weak roots case (*C*′_rl_ = 5 kPa) and a stronger roots case (*C*′_rl_ = 10 kPa). Shaded areas are defined by the upper and lower bound earth pressure solutions. Cohesion and cross-slope friction are invariant with slope. The net resistance on the downslope margin (i.e., downslope resistance-upslope drive) is always more than twice as large as the root cohesion, and becomes more important on shallower slopes.

While lateral root cohesion has been included in a few stability models for natural slopes [*Reneau and Dietrich*, 1987; *Montgomery et al*., 2000; *Gabet and Dunne*, 2002; *Casadei et al*., 2003a] lateral friction has generally been ignored. Figure 4 compares the lateral resistance due to cohesion and friction on a cross-slope margin and the net downslope resistance (i.e., resistance from the soil downslope of a block minus the driving stress from the soil upslope). The example shown in Figure 4 is for a block with a failure plane depth of 1 m, a friction angle of 40° and a saturation ratio of 0.5.

Friction on the cross-slope boundary provides ∽2 kPa of resistance, independent of the block's inclination (Figure 4). This suggests that cross-slope friction can be important in weakly rooted soils, as it is nearly half of the resistance provided by roots (Figure 4). Cross-slope friction is highly sensitive to failure plane depth (with a *z*^2^ dependence) but insensitive to friction angle (equation (7)). This is because as the friction angle increases, the earth pressure coefficient that controls the conversion from vertical to lateral stress decreases as 1 − sin*φ*′, while shear strength varies as normal stress multiplied by tan*φ*′. The product of these (tan*φ*′ (1 − sin*φ*′)) ranges from 0.26 to 0.30 for friction angles from 25° to 55° with its maximum at 38°.

Net downslope resistance is considerably larger than cross-slope resistance (Figure 4). It is most strongly dependent on cohesion but provides more strength than would be expected from cohesion alone; increasing cohesion by 5 kPa in Figure 4 increases net resistance by between 8 and 15 kPa. This amplified increase in resistance reflects the geometry of the upslope and downslope wedges. Since their failure surface is always longer than the failure depth, the additional strength is more than just the additional cohesion. Net downslope resistance also has a strong (*z*^2^) dependence on failure plane depth, a strong dependence on slope, a weak dependence on saturation ratio, and a negligible dependence on unit weight for both upper and lower bound solutions with resistance increasing with depth and unit weight but decreasing with slope angle and saturation ratio (equation (12)). Net resistance has a dependence on friction angle (not shown) that differs between the two formulations, increasing with friction angle in the lower bound case, and decreasing in the upper bound case. This reflects the influence of boundary friction (*δ*), which is assumed equal to soil friction angle (*φ*′) in the upper bound case, in reducing net resistance. The influence of boundary friction is absent from the lower bound case (i.e., *δ* = *θ*) so that net resistance increases with soil friction, reflecting the additional strength of the soil.

## 5. A Test of the Model

To test the model, we applied it to the highly instrumented Coos Bay (CB-1) slope that failed as a large debris flow in November 1996 [*Anderson et al*., 1997; *Montgomery et al*., 1997; *Torres et al*., 1998; *Montgomery et al*., 2009]. We chose this site because, whereas there remains some uncertainty over the geotechnical and hydrologic conditions appropriate for the site, the instrumentation at CB-1 provides one of the most comprehensive data sets in existence for a natural shallow landslide. At CB-1 we tested the model's ability to predict failure under the conditions measured during the 1996 storm, and whether the predicted failure was of a similar size to that which was observed.

### 5.1. Test Site Description

The CB-1 site, which was clear-cut in 1987, is located along Mettman Ridge approximately 15 km north of Coos Bay in the Oregon Coast Range. The hydrological behavior of the CB-1 experimental site was studied in detail over a period of 10 years [*Anderson et al*., 1997; *Montgomery et al*., 1997; *Torres et al*., 1998]. CB-1 is a 51 m long (860 m^2^) unchanneled valley with an average slope of 43°. The instrumentation at CB-1 included a grid of piezometers and tensiometers with continuous total head measurements from 1990 to the time of failure (in 1996). Piezometer records show that subsurface storm flow in the shallow, fractured-rock zone exerts the most significant control on pore pressure development in the CB-1 colluvium [*Montgomery et al*., 1997]. We use the piezometric surface at the time of slope failure estimated by *Montgomery et al*. [2009] from piezometers recording at the time of failure, but without any adjustment of the original pore pressure data.

The soil is well-mixed, nonplastic gravelly sand derived from weathered turbidite sandstone [*Schmidt et al*., 2001]. Low confining stress triaxial tests for samples from the site gave internal friction angles of 39.5° and 41° with effective soil cohesion of 0 to 1.8 kPa [*Montgomery et al*., 2009]. The soil bulk density (*ρ_s_*) ranges from 1200 to 1600 kg m^−3^ [*Schmidt et al*., 2001]. The soil thickness is well defined from soil borings [*Schmidt*, 1999]. *Montgomery et al*. [2009] measured basal and lateral root cohesions using the methods described by *Schmidt et al*. [2001]. They report a nonlinear decline in root cohesion with depth resulting in a spatially weighted average lateral root cohesion of 4.6 kPa and a basal cohesion of 0.1 kPa.

### 5.2. Method

**Figure 5:**
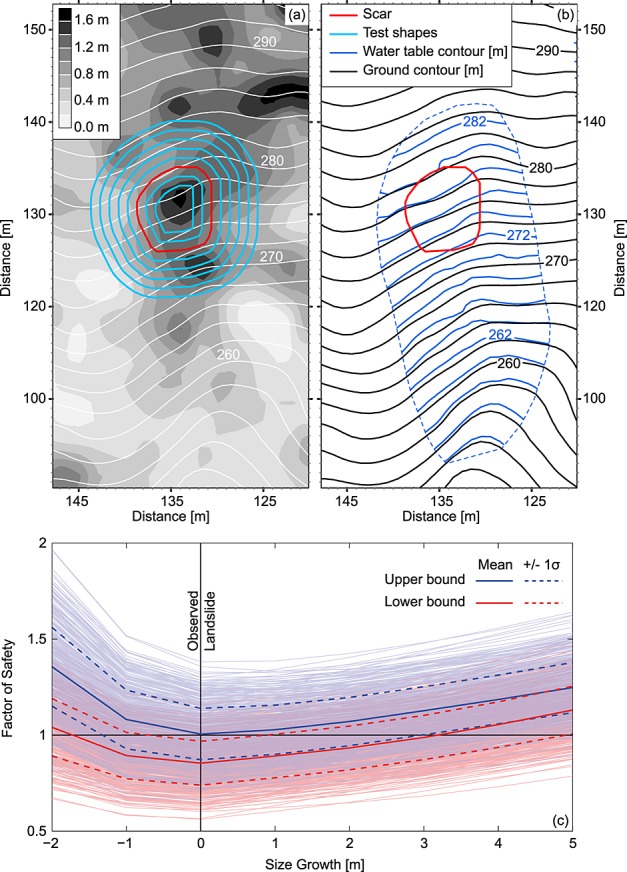
Model application to the CB-1 hillslope (Oregon, USA). (a) Map of the site showing the observed landslide scar (red), and the larger and smaller conformal shapes (blue) tested for stability. White contours show elevation (m), gray scale contours show soil depth (m). (b) Map showing elevation contours in black and piezometric surface contours in blue (m), soil unit weight (*γ_s_* = 15.7 kN m^−3^), friction angle (∽40°) and root cohesion (∽4 kPa) are also well constrained at the site. (c) The predicted factor of safety for the observed landslide (size growth = 0) and smaller and larger shapes generated by expanding and contracting the observed landslide geometry by a radial distance indicated on the *x* axis. Upper (blue) and lower (red) bounds are obtained using upper and lower bound earth pressure solutions. Pale lines show each of the 500 model runs described in section 5.2. thick dark lines show the mean FS from these runs ±1 standard deviation. Figures 5a and 5b are modified from *Montgomery et al*. [2009]. The model predicts failure for the observed scar geometry and finds that larger or smaller conformal shapes are more stable.

On the basis of these observations, we back calculate the stability of the observed landslide under a set of 500 feasible site conditions sampled from distributions to account for uncertainty in observed conditions at the site. For each prediction, we provide a lower bound on the stability estimate using the Rankine method and an upper bound using the Coulomb (upslope) and log-spiral (downslope) methods. We assume a spatially uniform soil density and sample from a uniform distribution with range 1200–1600 kg m^−3^ (unit weight = 15.7 kN m^−3^). We sample the friction angle from a normal distribution with mean 40° and standard deviation 2°; and the effective soil cohesion from a uniform distribution with the range 0–1.8 kPa. We use measured surface topography, soil depth, and pore water pressure data interpolated to a 1 m grid (Figures 5a and 5b). Topography and soil depth are very well constrained, we account for error in the pore water pressure data by uniformly introducing normally distributed error with a standard deviation of 10%. To represent the depth-varying lateral root cohesion we fit an exponential curve to the root cohesion with depth observations of *Montgomery et al*. [2009] from the CB-1 site, with the additional constraint that the average lateral root cohesion should be within ±0.1 kPa of the spatially weighted mean lateral root cohesion observed at the site. The best fit parameters within these constraints are *C*′_*r*0_ = 22 kPa and *j* = 4.96 m^−1^ (equation (20)); we sample these parameters from normal distributions using these mean values and standard errors of: 0.5 kPa and 0.73 m^−1^, respectively (ignoring covariance).

*Montgomery et al*. [2009] mapped the entire evacuated area at CB-1 and identified a smaller upper section of the failure, which they suggest was the initiation area on the basis of their onsite observations. Using the grid-based formulation of MD-STAB, we test the stability of this initiation area by using its geometry to define the group of potentially unstable columns in the stability model. To explore whether smaller or larger shapes would result in different outcomes, we shrink and expand the original shape by a constant distance around its perimeter and test their stability (Figure 5a).

### 5.3. Results

Figure 5c shows the factor of safety calculated from MD-STAB for the observed landslide geometry and a series of smaller and larger conformal shapes. Instability is confined within a range of sizes for these tested shapes. Shrinking the observed shape radially by 2 m or expanding it by more than 5 m results in stability in more than 95% of cases (defined by the different parameter sets). This sets limits on the possible size of the unstable area.

However, while most cases result in at least one stable shape many also predict at least one shape with FS < 1 (88% for lower bound and 51% for upper bound). This is not possible in reality since a landslide would already have initiated as soon as driving force exceeded resistance. In the CB-1 case a model run that predicts FS < 1 for any shape is likely associated with an unrealistically weak parameter set and a run that predicts FS > 1 for all shapes with an unrealistically strong set. Failure, with FS = 1, for the observed shape and no other is associated with an intermediate parameter set for the upper bound model and a high strength parameter set for the lower bound model (Figure 5c).

Of all the tested shapes, the observed landslide geometry is the least stable in 96% of cases. When size decreases the area-perimeter ratio also decreases, reducing both driving and basal resisting forces relative to the lateral resisting forces. When size increases under spatially variable conditions, the likelihood of including areas of increased strength also increases. We suggest that the interaction of these two effects defines an optimum, least stable, landslide geometry for a specific set of conditions. The CB-1 test shows that without any calibration MD-STAB produces stability predictions for this slope that are consistent with the observed landslide both in terms of its size and the conditions required for failure.

## 6. Discussion

### 6.1. Critical Depth and Area

Smaller patches with low-strength conditions are more likely than larger ones in a natural (heterogeneous) landscape, and thus, in the absence of any other control, the frequency of landslides should continuously increase with decreasing size [*Pelletier et al*., 1997; *Frattini and Crosta*, 2013; *Alvioli et al*., 2014]. Instead, many investigators have observed that there is a peak, or “rollover,” to the size-frequency distribution with fewer numbers of very small slides [e.g., *Hovius et al*., 1997; *Stark and Hovius*, 2001; *Malamud et al*., 2004; *Frattini and Crosta*, 2013]. We suggest that the minimum area that can fail under a given set of conditions (hereafter called the critical area) provides a mechanistic explanation of the infrequency of small landslides while the right tail is controlled by the size distribution of low-strength areas [*Pelletier et al*., 1997; *Katz and Aharonov*, 2006; *Frattini and Crosta*, 2013; *Alvioli et al*., 2014]. By setting FS equal to 1.0, equation (17) can be solved for the critical basal area *A_c_* at failure:


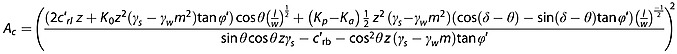
(24)

To explore how critical area changes with failure depth on a homogeneous slope, we examine a block with the material properties measured at CB-1 (friction angle = 40°, soil unit weight = 15.7 kN m^−3^, and exponential cohesion profile with *C*_0_ = 22 kPa and *j* = 4.96) and a slope angle of 36°, the average slope for the wider Coos Bay study area in which the landslide inventory shown in Figure 1 was collected [*Montgomery et al*., 2000].

**Figure 6:**
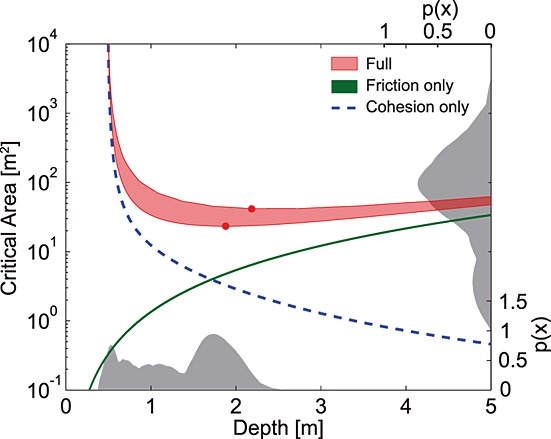
Critical area with depth for an equidimensional homogeneous block of soil at a slope of 36°, friction angle of 40°, *γ_s_* = 15.7 kN m^−3^ with a water table at the ground surface (i.e., fully saturated soil), assuming a l/*w* ratio of 1 (representative of the CB-1 scar). Note logarithmic *y* axis. Filled circles show the depths at which the critical area is minimized (*A_c_* = 23 m^2^ at *z* = 1.9 m in the lower bound case and *A_c_* = 42 m^2^ at *z* = 2.18 m in the upper bound case). Shaded areas are defined by the upper and lower bound earth pressure solutions; in the friction-only case these nearly coincide and the cohesion-only case does not have upper and lower bounds. The grey PDFs on the *x* and right axes show depth and area distributions respectively for 19 landslides in the Coos Bay catchment [*Montgomery*, 1991; *Larsen et al*., 2010].

When soil strength is provided entirely by friction, *A_c_* increases with depth from a minimum at the surface (Figure 6), whereas when it is provided entirely by cohesion *A_c_* decreases with depth from a maximum at the surface (note log scale on vertical axis). This is because the stability of a soil block is controlled by the relationship between its mass-dependent driving force and the resistance on its perimeter, both of which vary with failure depth. Driving force increases linearly with depth but friction resistance increases as the square of depth (*z*^2^ terms on top half of equation (24)), while root cohesion decreases exponentially with depth (equations (20) and (21)). When soil strength is provided by both friction and cohesion (“Full” lines in Figure 6), the interplay between the two components results in a range of depths with similar *A_c_*, and a critical depth that minimizes *A_c_* (indicated by filled circles in Figure 6). Although there is a range of depths that are close to critical, failure planes that are both shallower and deeper than this point are more stable and therefore require a larger *A_c_* for failure (Figure 6). This is true for both upper and lower bound solutions, which envelop the true value. These findings support those of *Dietrich et al*. [2008] and *Frattini and Crosta* [2013] that suggest a least stable depth, and imply that this least stable depth minimizes the critical area.

A critical depth in the range 0.5–3 m is consistent with observed shallow landslide depths (Figure 1c). A parameter exploration (not shown) suggests that increasing cohesion (by increasing *C*_0_ or by decreasing *j*) or friction (by decreasing *θ* or increasing *φ*′) results in a larger minimum critical area. However, increasing cohesion increases the depth at which the minimum critical area occurs, while increasing friction decreases it. Similar experiments (not shown) using uniform rather than depth-varying cohesion result in the same behavior but with an increase in the depth at which the minimum critical area occurs. This is because, when root cohesion is uniform, its contribution to basal resistance does not depend on depth, so its relative contribution to total resistance is very large at shallow depths and decreases rapidly with depth. Decreasing cohesion with depth simply enhances this effect. Figure 6 also shows that the difference between the upper and lower bound earth pressure solutions is large when cohesion is included and negligible when only friction is considered.

The critical area and the corresponding failure depth for this parameter set are in the range observed for shallow landslides (Figures 1a and 1c), and closely correspond to the modal landslide depth and area for landslides from the Coos Bay site on which the parameters have been based (Figure 6) [*Montgomery*, 1991; *Larsen et al*., 2010]. However, where soils are shallower than the critical depth, landslides will be very likely to fail at the soil-bedrock interface rather than within the harder bedrock. This is generally the case at Coos Bay, where most landslides failed at the soil-bedrock interface [*Montgomery et al*., 2000], which may explain the portion of observed Coos Bay failures with depths less than our prediction.

As noted above, the predicted critical depth and area can be close to zero in the case of a saturated cohesionless soil (Figure 6). This motivates the question: are there any constraints on critical depth and area for cohesionless soils? To address this we examine the behavior of a cohesionless block of soil 5 m long, 5 m wide, and 2 m deep, with a friction angle of 40° and a soil unit weight of 15.7 kN m^−3^. The slope angle is reduced from the average slope of the Coos Bay site to 30° to reflect the characteristics of cohesionless slopes. We test the stability of this block using equation (17) for slope-parallel failure planes at depths from 0.02 m to 10 m in increments of 0.01 m, beginning with an unsaturated block and increasing the water table height until failure occurs within the block.

**Figure 7:**
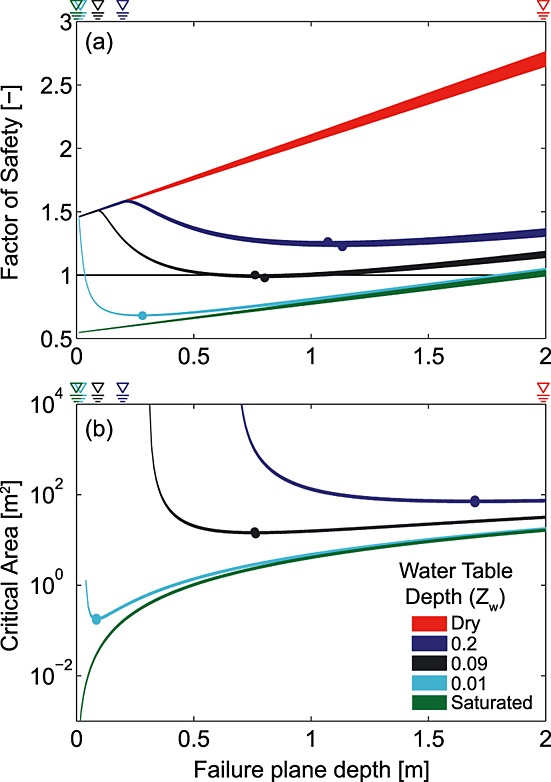
The factor of safety (a) and critical area (b) with depth for a block of soil where *θ* = 30°, *φ*′ = 40°, *γ_s_* = 15.7 kN m^−3^, for a range of water table depths (*Z_w_*). In both panels there are two lines for each water table depth, representing upper and lower bound solutions. The symbols above each plot indicate water table locations within the profile. Figure 7a shows the factor of safety for a 5 × 5 m block while Figure 7b shows the critical block area *A_c_*, which can vary. The dry case is stable at any area since tan*φ*′ > tan*θ*; thus, it has no critical area and does not appear in Figure 7b; the case of *z_w_* = 0.2 is stable for the 5 × 5 m block but appears in Figure 7b because it becomes unstable for critical areas >75 m^2^. Both factor of safety and critical area are minimized within the profile for partially saturated conditions.

Under dry conditions, the block is stable for all failure plane depths and FS increases linearly with depth (red curve in Figure 7a). This is because both the driving force and basal resistance increase linearly with depth, and lateral resistance increases as the square of depth (equation (17)). With a water table of 0.2 m below the ground surface or lower, the block remains stable at any depth (i.e., FS > 1) but there is a minimum FS within the profile (blue curves in Figure 7). When the failure plane is above the water table FS is the same as in the unsaturated case. Once the failure plane is below the water table, the saturated fraction of the soil column above the failure plane increases with failure plane depth causing FS to decrease. FS reaches a minimum at 1.2 m then begins to increase (blue curves in Figure 7a) because the lateral resistance increases at a higher rate compared to the reduction of shear resistance resulting from the increase of the saturated soil fraction (equation (24)). If the water table continues to rise, the block will fail at ∽0.8 m depth once the water table reaches 0.09 m depth (black curves in Figure 7a). As the water table approaches the surface, FS continues to decrease (cyan curve in Figure 7a).

Figure 7b shows that as the water table rises both the critical area and critical depth decrease. As the water table approaches the ground surface, the critical depth approaches zero and the critical area declines rapidly. When the water table reaches the ground surface the saturated fraction of the soil column no longer varies with depth, and the minimum FS is at the surface (green curve in Figure 7a) due to the more rapid increase of resisting force relative to driving force with depth (equation (24)). This explains why the critical failure plane depth and critical landslide size are both zero for cohesionless saturated soils (green curve in Figure 7b). Note that there is a critical area when *Z_w_* = 0.2 (*A_c_* = 75 m^2^), indicating that failure is possible at this water table depth but requires a much larger size than the 5 by 5 m block used in Figure 7a. The dry case is stable at any area since the slope is shallower than the friction angle and thus it has no critical area and does not appear in Figure 7b.

For a specific set of conditions, in a cohesive or cohesionless soil the water table height determines both the critical size and critical failure depth. Instability can occur when the area having that water table height expands to the critical size, or when a local increase of the water table sufficiently reduces the critical size. This suggests that the dynamics of the water table are an important control on landslide size and that topography exerts a strong control on landslide size not only through local slope but also through its influence on soil depth and water table height. These results also suggest that while cohesion leads to a minimum landslide size [*Reneau and Dietrich*, 1987; *Dietrich et al*., 2008; *Frattini and Crosta*, 2013], slide size is limited even in cohesionless landscapes. This provides a physical basis for a rollover in the landslide size distribution, albeit at a considerably smaller size than commonly reported [e.g., *Hovius et al.*,1997; *Stark and Hovius*, 2001; *Malamud et al*., 2004; *Stark and Guzzetti*, 2009].

### 6.2. Critical Shape

**Figure 8:**
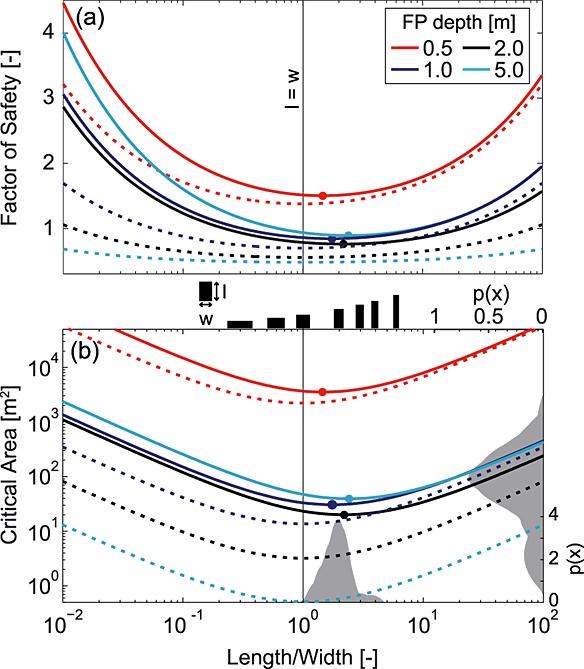
The (a) factor of safety and (b) critical area as a function of length-width (*l*/*w*) ratio for a block of soil with *θ* = 36°, *φ*′ = 40°, and *γ_s_* = 15.7 kN m^−3^, with a water table at the ground surface (i.e., fully saturated soil) and where resistance is provided by cohesion only (dashed lines) and both friction and cohesion (solid lines). Filled circles indicate *l*/*w* ratios that minimize FS and critical area for each depth. In Figure 8a the block area is held constant at 60 m^2^ (representative of the CB-1 scar) to calculate FS. In Figure 8b the grey PDFs on the *x* and right axes show *l*/*w* ratio and area distributions, respectively, for 19 landslides in the Coos Bay catchment [*Montgomery*, 1991; *Larsen et al*., 2010]. The black rectangles between Figures 8a and 8b are illustrative of the *l*/*w* ratios corresponding to their *x* axis location. Least stable shapes are equidimensional considering only cohesion but longer than they are wide once friction is included.

While it is commonly observed that landslide length exceeds width [*Gabet and Dunne*, 2002; *Rickli et al*., 2008; *Marchesini et al*., 2009], this behavior has not been fully explained. In a second set of experiments using the saturated cohesive scenario (*θ* = 36°, *φ*′ = 40°, *γ_s_* = 15.7 kN m^−3^, *C*_0_ = 22 kPa, *j* = 4.96), we explore the impact of shape (in terms of the length-width ratio) on FS and critical area of a potential landslide. We calculate FS and critical area of blocks of depth 0.5, 1, 2, and 5 m, varying the length-width ratio from 0.01 to 100 to find the ratio that minimizes FS and critical area (Figure 8). Here we show only results using the lower bound earth pressure formulation (i.e., Rankine's method), which is conservative in terms of its predicted FS, critical area and depth because it predicts shallower failures with a smaller minimum area. Results (not shown) using the upper bound earth pressure formulation generally exhibit similar behavior, but differ slightly in their absolute values, due to the increased resistance on the upslope and downslope margins.

The least stable shape is that which minimizes resisting force relative to driving force. When lateral strength is provided by cohesion alone (dashed lines in Figure 8) the least stable shape is equidimensional (i.e., *l*/*w* = 1 minimizes FS and *A_c_*) independent of block depth, because this minimizes perimeter length for a given area. Once a friction component is introduced, resistance on the upslope and downslope margins dominates (Figure 4), and scales with the cross-sectional area of these margins. On natural slopes failure depth is limited by soil depth (typically to a maximum of a few meters). Width is thus the main control on the cross-sectional area of the upslope and downslope margins, leading to wider shapes having a higher FS for a given area (Figure 8a) or a larger critical area (Figure 8b). FS and critical area increase again when *l*/*w* ratio is greater than 3 as the perimeter to area ratio is then large enough to overcome the effect of the strength difference between the margins.

The least stable shapes (marked with filled circles in Figure 8) are consistently longer than they are wide. The least stable *l*/*w* ratio increases with increasing block depth from 1.5 to 5, due to the increased strength on the downslope margin. This is because, as depth increases, a greater fraction of the resistance is provided by friction, and the strength on the downslope boundary becomes more important. A parameter exploration (not shown) suggests that length exceeds width for all parameter combinations except when the slope exceeds the friction angle.

These results imply that for similar size low-strength patches, the patch that is oriented with its long axis downslope should be less stable. This is consistent both with the general observation that shallow landslide scars are longer than they are wide (Figure 1b) and with the *l*/*w* ratios of landslides observed in the Coos Bay catchment, which was used to parameterize the model (Figure 8b) [*Montgomery*, 1991]. However, it is unlikely that the strength difference between the downslope and cross-slope margins is the sole reason for this pattern. The shape of an unstable patch is controlled by the spatial pattern of the driving parameters (particularly, pore water pressure and soil depth), which is not random, but rather is strongly controlled by topography and often oriented with greater values in the downslope direction.

### 6.3. Depth-Area Scaling

**Figure 9:**
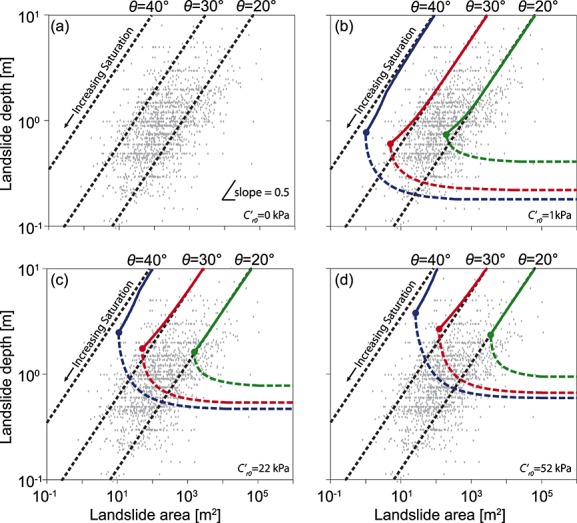
Grey diamonds show landslide scar depth and area for a global compilation of soil landslides [*Larsen et al*., 2010]. Colored lines show the modeled relationship between failure plane depth and critical area for slopes with *θ* = 20°, 30°, and 40°, *φ*′ = 40°, and *γ_s_* = 15.7 kN m^−3^. Different panels reflect different root cohesion scenarios: (a) *C′*_*r*0_ = 0, (b) *C*′_*r*0_ = 1, (c) *C*′ _*r*0_ = 22 (the CB-1 value), and (d) *C*′_*r*0_ = 52 kPa (representing old growth forest). In every case *j* = 4.96 kPa^−1^. Solid lines indicate the relationship between critical depth and area when neither are constrained, with filled circles indicating where these are minimized. Dashed lines represent the relationship for a saturated soil where depth is limited by soil depth. The model predicts a theoretical lower limit to landslide area given depth, and the *θ* = 40° curve is a lower bound on the observed scar areas. When landslide depth becomes limited by soil depth, critical area increases as depth decreases, creating a theoretical lower limit on landslide area for a given depth.

Finally, we explore the relationship between critical area and the depth that minimizes that area. We perform a set of numerical experiments where soil unit weight and friction angle are held constant at the values measured at CB-1. We test a range of slopes with different combinations of slope angle (*θ* = 20°, 30°, and 40°), root cohesion (*C*′_*r*0_ = 0, 1, 22, and 52 kPa; *j* = 4.96 m^−1^) and water table depth (0–10 m in 0.02 m increments). These conditions represent typical ranges for landscapes in which shallow landslides occur. For each combination, failure planes are tested (in 0.02 m increments) from the surface to the base of the soil column to find the minimum critical area and record its corresponding depth. For simplicity, only the lower bound solutions (i.e., from Rankine's method) are shown in Figure 9. The upper bound solutions (using Coulomb and log-spiral methods) result in slightly larger critical areas.

The curves in Figure 9 show critical area and depth for slopes with the same material properties but varying saturation, for different cohesion scenarios. They are compared to a global compilation of observations from *Larsen et al*. [2010]. In the cohesionless case there is an approximately square root relationship between critical area and depth (Figure 9a). All but one of the observations have scar areas that exceed those defined by the 40° curve. When cohesion is introduced, critical area and depth both decrease with increasing saturation following a similar square root relationship to a lower limit at fully saturated conditions (filled circles in Figures 9^9^b–9^9^d). However, on natural slopes soil depth is often less than a few meters and many landslides have their failure plane at the base of the soil [e.g., *Montgomery et al*., 2000]. When landslide depth is limited by soil depth (as suggested by *Larsen et al*. [2010]), the failure surface is forced to the base of the soil column and critical area increases as soil depth decreases (dashed colored lines in Figures 9b–9^9^d). This is because the resistance due to root cohesion becomes increasingly dominant relative to the driving force. The dashed colored lines in Figure 9 represent the minimum critical area occurring under fully saturated conditions. Reducing saturation results in an increase of critical area at a given depth (not shown).

Varying the slope angle has a strong impact on the coefficient but a weak impact on the exponent of the depth-area relationship, suggesting similar scaling behavior independent of the material properties. The three cohesion scenarios shown in Figures 9b–9d encompass conditions from weak grassland to strong natural forest root networks. When cohesion is low (Figure 9b), the depth-area curve provides a lower bound to the observations. Increasing cohesion results in curves that encompass progressively fewer observations (Figures (9)9c–9^9^d). Our results show that increasing cohesion increases both the minimum landslide depth and critical area, suggesting that in landscapes with stronger cohesion landslides should be both larger and deeper consistent with observations [e.g., *Selby*, 1976; *Gabet and Dunne*, 2002].

The roughly square root dependence of depth on area is consistent with the observations; the best fit for observed soil landslides yields an exponent of 0.4 [*Larsen et al*., 2010]. In cohesionless soils, the predicted exponent is always 0.5 and equation (24) can be rearranged to solve for depth in terms of critical area:


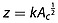
(25)

where


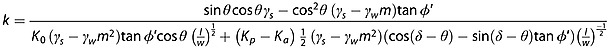
(26)

When cohesion is introduced, the lateral resistance becomes a more complex function of depth and thus the exact relationship becomes dependent on the specific conditions, how cohesion is parameterized, and the relative importance of friction and cohesion.

The modeled depth-area curves represent the critical failure plane depth and the minimum landslide area for a given set of conditions. Our findings differ from those of *Klar et al*. [2011] in that we suggest that depth only imposes a lower bound on size, whereas they suggested that area defines depth. As a result our model only explicitly explains the trend in observed minimum landslide area with depth, which is well captured by the *θ* = 40° curve in Figure 9b. However, since smaller low-strength patches are likely to be more common in a natural (heterogeneous) landscape [*Pelletier et al*., 1997; *Frattini and Crosta*, 2013], we might expect landslide areas to cluster near their lower size limit, explaining the similar trend in maximum landslide area for a given depth with the majority of the data plotting between the modeled 20° and 30° curves (Figure 9). We suggest that our model is an alternative explanation of the observed landslide depth-area scaling to that of *Klar et al*. [2011], both based on limit equilibrium slope stability models. Since Klar et al. find the depth-area scaling from experiments in which length is constrained, their approach might suggest that landslide area is set first (e.g., by slope length or the area of a low-strength patch) and that landslide depth is then dependent on this area. Since we find the critical (or minimum) landslide area for a given landslide depth, our approach might suggest that depth is set first (e.g., by pore pressure or soil depth) and that landslide area is then dependent on depth. Both situations are conceivable on natural slopes and it is interesting that both approaches result in approximately square root relationships between landslide depth and area.

### 6.4. Model Assumptions and Requirements

MD-STAB is a shallow landslide slope stability model and as such is limited in its application to failures within or at the base of the soil. In common with most other shallow landslide models, our model assumes that failure occurs under drained conditions. This is appropriate for the colluvial soils found on many natural slopes but not for clay-rich materials.

Our model also assumes hydrostatic conditions in the calculation of active and passive pressures on the upslope and downslope margins of the central block. In reality slope-parallel seepage will alter these pressures but we currently lack methods that account for them. A reduction in net downslope resistance due to seepage forces would slightly reduce critical area, increase optimum depth, and make the least stable shape slightly rounder. However, this does not alter our general findings that (1) there is a critical area and minimum depth for both cohesive and cohesionless soils, (2) blocks that are longer than they are wide are least stable, and (3) critical area scales as the square of optimum depth under most conditions found in natural landscapes.

*Jaky*'s [1944] empirical formula, which assumes cohesionless soil, may overestimate the cross-slope earth pressure coefficient. However, resistance due to at-rest earth pressure on the cross-slope boundary is small relative to other components (Figure 4), so small changes to the value of *K*_0_ will have little impact on the net resistance. To assess the potential impact of this assumption, we tested the extreme case of neglecting the cross-slope earth pressure term (i.e., *K*_0_ = 0) and found that our results show very little sensitivity to the value of this coefficient. Moreover, changes in *K*_0_ do not alter the linear dependence of at-rest earth pressure on depth, which drives our findings on optimum depth and depth-area scaling.

In our model the landslide is assumed to have a parallelepipedal shape, with vertical sides. The assumption that cross-slope margins are vertical rather than inclined or curved will minimize their surface area and resulting resistance [*Stark and Eid*, 1998]. This is consistent with field observations, which suggest that the head scarps of shallow landslides are generally near-vertical and that their cross-slope margins are also steep. Failure geometry at the downslope boundary is poorly constrained by observations, because of subsequent erosion following failure. Nevertheless, where observation has been possible, a low-angle failure surface generally connects the ground surface with the basal failure plane [*Milledge*, 2008], consistent with the wedge representation used here. The assumption that the failure plane is parallel to the ground surface is reasonable for shallow translational landslides where the radius of curvature of the failure surface is typically very low, and enables us to limit the search space for critical failure depth to one dimension.

In MD-STAB the potential failure mass is treated as a rigid block although in reality a failure may occur progressively if small-scale cracks coalesce into a continuous failure plane [*Petley et al*., 2005] or locally high strain induces liquefaction [*Iverson et al*., 2000]. At present representing such progressive failure is generally limited to computationally intensive continuum methods, although *Lehmann and Or* [2012] have developed an innovative approach to represent this progressive failure implicitly. We have applied our boundary force equations within a limit equilibrium framework to examine their implications for landslide size and shape. However, we note that our equations could easily be applied within a framework similar to that of *Lehmann and Or* [2012], which would account for the forces acting on the margins due to the self-weight of the soil and would result in a more appropriate method for natural slopes.

The parameters required to run MD-STAB are the same as those required to evaluate the infinite slope equation: surface slope and friction angle, soil cohesion and unit weight, failure plane depth, and water table height. Several of these parameters are either derived from or strongly influenced by topography; for example, local slope, soil depth, and pore water pressure could be modeled in a similar way to *Dietrich et al*. [1995, 2008]. Other parameters are likely to vary in space, but the magnitude and correlation length of their variability are unknown in most landscapes so that they are generally assumed spatially uniform as we have done here. Cohesion due to roots is likely to vary with depth below the surface. There is reasonable observational support for an exponential relationship between root cohesion and depth in many landscapes [*Hales et al*., 2009] enabling root cohesion to be simply represented with the addition of only one parameter. However, MD-STAB is not bound to this particular representation, requiring only a root strength field. Similarly, we have chosen a very simple representation of pore water pressure (assuming steady slope-parallel flow), but more complex alternatives that provide a pore pressure field could be utilized. The only additional data requirement for MD-STAB is the identification of cells that are within the shape whose stability is to be tested. However, this is a key barrier to the model's application to a discretized landscape. While stability can be calculated analytically for each potential landslide, testing all possible combinations of cells would be exponentially complex; the number of tests goes as 2^(nrows × ncols)^ or 10,000 combinations for a 10 by 10 cell grid. In forthcoming papers this model is coupled with a novel search algorithm to predict landslides across a landscape (D. Bellugi et al., A spectral clustering search algorithm for predicting shallow landslide size and location, submitted to *Journal of Geophysical Research: Earth Surface*, 2014a; D. Bellugi et al., Predicting shallow landslide size and location across a natural landscape: Application of a spectral clustering search algorithm, submitted to *Journal of Geophysical Research: Earth Surface,* 2014b).

## 7. Conclusion

In this paper we derive MD-STAB, a new multidimensional shallow slope stability model, which predicts that observed shallow landslide depth-area scaling in both cohesive and cohesionless soils arises from depth-varying friction on the margins of a potential landslide. MD-STAB accounts for the forces acting on all boundaries of a potential landslide and is statically determinate. It represents lateral root cohesion and earth pressure on inclined slopes, making it suitable for natural landscapes. This model is easily applied to spatially gridded data, requires only a modest parameterization (i.e., the same as the infinite slope) and is therefore suitable for landscape-scale application.

MD-STAB successfully predicts the failure of a well-documented shallow landslide in which measured parameters, including pore pressure and root strength, are used. The model also predicts that larger or smaller shapes conformal to that observed are indeed more stable. For smaller shapes stability is due to the increased influence of resistance on the margins, whereas for larger shapes stability is due to the inclusion of areas of increased strength.

We explore the influence of lateral friction and cohesion on slope stability and landslide scale (depth and area) using an inclined block of soil with fixed strength parameters but varying pore pressure and soil depth. Lateral friction on the boundaries of a potential landslide increases considerably the magnitude of lateral reinforcement. Friction and cohesion interact to create a critical depth at which shallower and deeper potential failure planes are more stable. This critical depth develops even in cohesionless soils when they are less than fully saturated. As a result, landslides should have a minimum area for failure in both cohesive and cohesionless soils. Friction and cohesion also impose a least stable shape that is longer than it is wide, even in homogeneous hillslope conditions. Minimum scar area is predicted to increase as approximately the square of failure plane depth, consistent with, and bounding, observed landslide depth-area data.

These findings suggest that a peak, or rollover, in observed landslide size-frequency distributions should be expected, and that the observed depth-area scaling is related to the depth-varying lateral frictional resistance. We hypothesize that the right tail of observed landslide size-frequency distributions is controlled by the heterogeneity of local conditions. Exploring this hypothesis will require applying this model to real landscapes to determine size and location of landslides under a variety of conditions.

## APPENDIX

### Appendix A

Below are the equations for the log-spiral earth pressure representation for sloping soils that include both friction and cohesion strength following *Soubra and Macuh* [2002].

(A1)

(A2)

(A3)

(A4)

(A5)

(A6)

(A7)

(A8)

(A9)

(A10)

**Notation:**

Variable	Units	Description
*A_c_*	m^2^	critical basal area of the central block required for failure
*C*′_*r*0_	Pa	coefficient representing the maximum root cohesion value at the surface
*C*′_rb_	Pa	root cohesion on the basal failure surface
*C*′_rl_	Pa	depth averaged lateral root cohesion
*F*_dc_	N	central block driving force
*F*_du_	N	slope-parallel component of the active force
*F*_nc_	N	normal force central block weight force acting on the failure surface
*F*_nd_	N	slope normal component of the passive force
*F*_nt_	N	total normal force acting on the basal failure surface of the central block
*F*_nu_	N	slope normal component of the active force (negative for a net resisting force) on the upslope margin of the central block
*F_p_*	N	passive force
*F*_rc_	N	resisting force on each cross-slope side of the slide block
*F*_rd_	N	slope-parallel component of the passive force
*FS*	-	factor of safety
*F_w_*	N	central block weight force
*g*	m s^−2^	gravitational acceleration
*h*	m	water table height above failure surface
*j*	m^−1^	*e*-folding length scale for root cohesion with depth in the soil profile
*K*_0_	-	coefficient of at-rest earth pressure
*K_a_*	-	active earth pressure coefficient
*K*_ah_	-	horizontal active earth pressure coefficient
*K_p_*	-	passive earth pressure coefficient
*K*_ph_	-	horizontal passive earth pressure coefficient
*K*_pc_	-	cohesion component of *Soubra and Macuh*'s [2002] passive earth pressure coefficient
*K_pγ_*	-	friction component of *Soubra and Macuh*'s [2002] passive earth pressure coefficient
*l*	m	true downslope length of the slide block
*m*	-	saturation ratio
*s_c_*	Pa	resisting stress on the cross-slope sides of the slide block
*w*	m	cross-slope width of the slide block
*x_c_*	m	cross-slope planimetric coordinate
*y_c_*	m	downslope planimetric coordinate
*z_c_*	m	vertical coordinate
*z*	m	failure surface depth below the ground surface
*z_w_*	m	water table depth below the ground surface
*α*_0_, *α*_1_	°	geometry parameters for the logarithmic-spiral failure surface
*β*	°	inclination from horizontal of failure plane from base of central block to ground surface upslope
*δ*	°	boundary friction angle
*γ_s_*	N m^−3^	unit weight of the soil
*γ_w_*	N m^−3^	unit weight of water
*ϕ*′	°	soil friction angle
*ρ_s_*	kg m^−3^	bulk density of soil
*σ*′*_x_*	Pa	at-rest lateral earth pressure
*σ*′*_z_*	Pa	vertical effective pressure
*σ_a_*	Pa	active stress on the upslope margin of the central block
*σ_p_*	Pa	passive stress on the downslope margin of the central block
*σ_z_*	Pa	total vertical geostatic stress
*θ*	°	slope inclination
*τ*	Pa	driving stress

## Key Points

- We derive a multidimensional stability model appropriate for natural landscapes
- In all soils there is a critical depth and a minimum size for shallow landslides
- The model provides a mechanistic explanation for observed depth-area scaling
